# Supplementary material for: NeoMUST: an accurate and efficient multi-task learning model for neoantigen presentation
Source: Life Sci Alliance. 2024 Jan 30;7(4):e202302255. doi: 10.26508/lsa.202302255 (PMC10828515; doi:10.26508/lsa.202302255)
Supplement: Supplementary file 2 [file LSA-2023-02255_TableS1.docx]

Content

[1 Supplementary Table 1 2](#_Toc10662)

[2 Supplementary Table 2 3](#_Toc31233)

[3 Supplementary Table 3 5](#_Toc5071)

[4 Supplementary Table 4 7](#_Toc29047)

[5 Supplementary Table 5 9](#_Toc13368)

[6 Supplementary Table 6 11](#_Toc506)

[7 Supplementary Table 7 13](#_Toc6931)

[8 Supplementary Table 8 15](#_Toc15200)

[9 Supplementary Table 9 16](#_Toc32668)

[10 Supplementary Table 10 17](#_Toc19372)

# 1 Supplementary Table 1

| Test Sets/Metrics | Statistics | MHCflurry2.0 BA | MHCflurry2.0 PS | NetMHCpan4.0 EL | NeoMUST NP |
| --- | --- | --- | --- | --- | --- |
| TeSet-1/PPV | Mean | 0.354272356 | 0.403863331 | 0.363439693 | 0.402376727 |
|  | Median | 0.37455827 | 0.418704471 | 0.394973212 | 0.438678247 |
|  | Upper quartile | 0.445416842 | 0.490987729 | 0.475133512 | 0.502359029 |
|  | Lower quartile | 0.243270817 | 0.332670455 | 0.267610264 | 0.31702689 |
|  | P Value | 4.96E-06 | 6.19E-01 | 4.28E-03 |  |
| TeSet-1/AUC-ROC | Mean | 0.907501453 | 0.91430812 | 0.892871274 | 0.913866985 |
|  | Median | 0.917354697 | 0.926337946 | 0.910767169 | 0.922050338 |
|  | Upper quartile | 0.952585906 | 0.9570316 | 0.951222268 | 0.963882549 |
|  | Lower quartile | 0.882458538 | 0.894211835 | 0.861470556 | 0.886429717 |
|  | P Value | 1.86E-03 | 8.60E-01 | 1.18E-09 |  |
| TeSet-1/AUC-PR | Mean | 0.271897921 | 0.360626399 | 0.309699156 | 0.341626537 |
|  | Median | 0.275911739 | 0.365407823 | 0.329515236 | 0.357081321 |
|  | Upper quartile | 0.366907589 | 0.445251368 | 0.425356509 | 0.4618114 |
|  | Lower quartile | 0.144438494 | 0.265457481 | 0.192506024 | 0.226482818 |
|  | P Value | 4.57E-08 | 0.040341831 | 9.86E-02 |  |
| TeSet-2/PPV | Mean | 0.595660434 |  | 0.588921517 | 0.601011687 |
|  | Median | 0.612959948 |  | 0.611399454 | 0.62152833 |
|  | Upper quartile | 0.689193469 |  | 0.682793215 | 0.699879903 |
|  | Lower quartile | 0.522351092 |  | 0.519984016 | 0.538531458 |
|  | P Value | 2.56E-01 |  | 2.14E-02 |  |
| TeSet-2/AUC | Mean | 0.957487212 |  | 0.952907822 | 0.960564573 |
|  | Median | 0.97292824 |  | 0.970863524 | 0.975356854 |
|  | Upper quartile | 0.981390742 |  | 0.982282064 | 0.982433698 |
|  | Lower quartile | 0.947425287 |  | 0.946637227 | 0.957050067 |
|  | P Value | 0.001435943 |  | 1.92E-06 |  |
| TeSet-2/AUC-PR | Mean | 0.591082685 |  | 0.593218843 | 0.603159502 |
|  | Median | 0.629790522 |  | 0.630671438 | 0.63741742 |
|  | Upper quartile | 0.703291881 |  | 0.696043565 | 0.732422909 |
|  | Lower quartile | 0.49956374 |  | 0.488237454 | 0.494489225 |
|  | P Value | 0.049613243 |  | 6.53E-02 |  |

**Supplementary Table 1. Statistics of Performance Metrics to Compare NeoMUST to other Models on TeSet-1 and TeSet-2.**
