# Supplementary material for: NeoMUST: an accurate and efficient multi-task learning model for neoantigen presentation
Source: Life Sci Alliance. 2024 Jan 30;7(4):e202302255. doi: 10.26508/lsa.202302255 (PMC10828515; doi:10.26508/lsa.202302255)
Supplement: Supplementary file 3 [file LSA-2023-02255_TableS2.docx]

# 2 Supplementary Table 2

| HLA | NetMHCpan4.0 EL | MHCflurry2.0 BA | MHCflurry2.0 PS | NeoMUST NP |
| --- | --- | --- | --- | --- |
| HLA-A*01 | 0.851470547 | 0.859305039 | 0.86720674 | 0.858094345 |
| HLA-A*02 | 0.875567732 | 0.879517257 | 0.896050028 | 0.88504178 |
| HLA-A*03 | 0.818344943 | 0.814151559 | 0.82680164 | 0.816391131 |
| HLA-A*11 | 0.815881196 | 0.811290869 | 0.85214634 | 0.81942407 |
| HLA-A*23 | 0.938314215 | 0.932668346 | 0.951901039 | 0.958749702 |
| HLA-A*24 | 0.931673144 | 0.934460524 | 0.948101877 | 0.921518918 |
| HLA-A*25 | 0.918751013 | 0.946988567 | 0.960553402 | 0.936292934 |
| HLA-A*26 | 0.895314434 | 0.911624558 | 0.932997097 | 0.913944628 |
| HLA-A*29 | 0.836552068 | 0.81121141 | 0.865097943 | 0.808380397 |
| HLA-A*30 | 0.878846011 | 0.900655542 | 0.915895727 | 0.914832666 |
| HLA-A*31 | 0.803294391 | 0.837430319 | 0.857237389 | 0.86485463 |
| HLA-A*32 | 0.835677377 | 0.844170212 | 0.895310225 | 0.853071117 |
| HLA-A*68 | 0.914571258 | 0.926598448 | 0.938079661 | 0.942319064 |
| HLA-B*07 | 0.884348331 | 0.882803067 | 0.885754868 | 0.890270075 |
| HLA-B*08 | 0.922640805 | 0.935581563 | 0.937361453 | 0.923371982 |
| HLA-B*13 | 0.828595743 | 0.823379099 | 0.890491708 | 0.830616925 |
| HLA-B*14 | 0.884274718 | 0.929612604 | 0.961530709 | 0.957639494 |
| HLA-B*15 | 0.919463814 | 0.929503137 | 0.939734377 | 0.932436439 |
| HLA-B*18 | 0.868602691 | 0.886508112 | 0.907848909 | 0.893475034 |
| HLA-B*27 | 0.802856091 | 0.807881186 | 0.817382533 | 0.760346677 |
| HLA-B*35 | 0.92388231 | 0.929173222 | 0.941629844 | 0.925946417 |
| HLA-B*37 | 0.923997189 | 0.952478025 | 0.958239448 | 0.968545032 |
| HLA-B*38 | 0.942436814 | 0.963597842 | 0.972672756 | 0.966011243 |
| HLA-B*39 | 0.868430275 | 0.917585463 | 0.911027268 | 0.912560411 |
| HLA-B*40 | 0.882775651 | 0.899372446 | 0.904629168 | 0.90005862 |
| HLA-B*41 | 0.716764706 | 0.884901961 | 0.862156863 | 0.961372549 |
| HLA-B*44 | 0.891448473 | 0.87910295 | 0.877725733 | 0.892223989 |
| HLA-B*45 | 0.897396656 | 0.917595134 | 0.902606911 | 0.937109027 |
| HLA-B*47 | 0.809982739 | 0.829545455 | 0.800920598 | 0.753883774 |
| HLA-B*50 | 0.914685877 | 0.936715065 | 0.946561161 | 0.952751761 |
| HLA-B*51 | 0.828025029 | 0.832989929 | 0.873782339 | 0.844953474 |
| HLA-B*55 | 0.920812447 | 0.937529556 | 0.960906712 | 0.942384691 |
| HLA-B*56 | 0.844716469 | 0.907273948 | 0.898118443 | 0.902496697 |
| HLA-B*57 | 0.861125341 | 0.853691904 | 0.853515728 | 0.856014085 |
| HLA-B*58 | 0.894406073 | 0.880623483 | 0.931180387 | 0.836926762 |
| HLA-B*73 | 0.503326996 | 0.794647523 | 0.782053592 | 0.858679248 |
| HLA-C*01 | 0.859502609 | 0.865752037 | 0.913283098 | 0.83581393 |
| HLA-C*02 | 0.764928413 | 0.762503731 | 0.808391411 | 0.810248114 |
| HLA-C*03 | 0.883388184 | 0.894581263 | 0.910465369 | 0.892135974 |
| HLA-C*04 | 0.844805126 | 0.807461676 | 0.87485304 | 0.795227717 |
| HLA-C*05 | 0.84760309 | 0.827337199 | 0.864752799 | 0.818763029 |
| HLA-C*06 | 0.925841503 | 0.941254088 | 0.950619147 | 0.943774086 |
| HLA-C*07 | 0.834951373 | 0.83892398 | 0.870271609 | 0.850610924 |
| HLA-C*08 | 0.860235386 | 0.882392142 | 0.904200427 | 0.864459006 |
| HLA-C*12 | 0.862863161 | 0.865280304 | 0.895754441 | 0.876312023 |
| HLA-C*14 | 0.949280144 | 0.953516823 | 0.973295957 | 0.959812593 |
| HLA-C*15 | 0.583333333 | 0.833333333 | 0.875 | 0.75 |
| HLA-C*16 | 0.814072932 | 0.811454682 | 0.828919902 | 0.82145247 |

**Supplementary Table 2. Means of AUC-ROCs for Different Alleles in TeSet-1.** The means were calculated for all MHC-1 molecules sharing the same gene and allelic group, e.g. HLA-A*02.
