# Supplementary material for: NeoMUST: an accurate and efficient multi-task learning model for neoantigen presentation
Source: Life Sci Alliance. 2024 Jan 30;7(4):e202302255. doi: 10.26508/lsa.202302255 (PMC10828515; doi:10.26508/lsa.202302255)
Supplement: Supplementary file 4 [file LSA-2023-02255_TableS3.docx]

# 3 Supplementary Table 3

| HLA | NetMHCpan4.0 EL | MHCflurry2.0 BA | MHCflurry2.0 PS | NeoMUST NP |
| --- | --- | --- | --- | --- |
| HLA-A*01 | 0.551256829 | 0.525295513 | 0.519532942 | 0.52852409 |
| HLA-A*02 | 0.425501046 | 0.29766043 | 0.494299493 | 0.428480807 |
| HLA-A*03 | 0.400019013 | 0.327424034 | 0.370010679 | 0.320831883 |
| HLA-A*11 | 0.31766212 | 0.302153809 | 0.316331498 | 0.312260598 |
| HLA-A*23 | 0.789163961 | 0.847605519 | 0.925933442 | 0.865868506 |
| HLA-A*24 | 0.619328524 | 0.643270143 | 0.696774134 | 0.594658668 |
| HLA-A*25 | 1 | 0.909090909 | 0.909090909 | 1 |
| HLA-A*26 | 0.97826087 | 0.97826087 | 0.986111111 | 0.97826087 |
| HLA-A*29 | 0.238709677 | 0.132258065 | 0.132258065 | 0.065591398 |
| HLA-A*30 | 0.6625 | 0.575 | 0.6625 | 0.6875 |
| HLA-A*31 | 0.524509804 | 0.443627451 | 0.587009804 | 0.484068627 |
| HLA-A*32 | 0.633063508 | 0.480535061 | 0.683087566 | 0.559783037 |
| HLA-A*68 | 0.716916695 | 0.666418047 | 0.806657088 | 0.769929006 |
| HLA-B*07 | 0.585012469 | 0.568010646 | 0.573727346 | 0.551681105 |
| HLA-B*08 | 0.713161585 | 0.738403221 | 0.660634077 | 0.663783564 |
| HLA-B*13 | 0.622596643 | 0.7217375 | 0.83619818 | 0.834765757 |
| HLA-B*14 | 0.548701299 | 0.533841159 | 0.575799201 | 0.545204795 |
| HLA-B*15 | 0.783843504 | 0.799146629 | 0.863297899 | 0.861386587 |
| HLA-B*18 | 0.543646508 | 0.5964127 | 0.564491518 | 0.481695959 |
| HLA-B*27 | 0.06757757 | 0.10573288 | 0.097310964 | 0.020902463 |
| HLA-B*35 | 0.777943065 | 0.77724671 | 0.814706752 | 0.768830057 |
| HLA-B*37 | 0.628571429 | 0.542857143 | 0.685714286 | 0.657142857 |
| HLA-B*38 | 0.755166508 | 0.827697208 | 0.877541854 | 0.841130167 |
| HLA-B*39 | 0.784640693 | 0.929731602 | 0.877419913 | 0.945748918 |
| HLA-B*40 | 0.500753608 | 0.515435478 | 0.574529687 | 0.554172267 |
| HLA-B*41 | 0 | 0.5 | 0.5 | 1 |
| HLA-B*44 | 0.542483477 | 0.503621369 | 0.520611673 | 0.530436836 |
| HLA-B*45 | 0.972222222 | 0.819444444 | 0.916666667 | 0.972222222 |
| HLA-B*47 | 1 | 1 | 1 | 1 |
| HLA-B*50 | 0.875331565 | 0.89071618 | 0.934748011 | 0.865782493 |
| HLA-B*51 | 0.259090909 | 0.259090909 | 0.295454545 | 0.504545455 |
| HLA-B*55 | 0.571428571 | 1 | 0.857142857 | 0.714285714 |
| HLA-B*56 | 0.541666667 | 0.541666667 | 0.819444444 | 0.361111111 |
| HLA-B*57 | 0.574541713 | 0.470304901 | 0.548681257 | 0.49822297 |
| HLA-B*58 | 0.4 | 0.4 | 0.6 | 0.5 |
| HLA-B*73 | 0.642857143 | 0.785714286 | 0.785714286 | 0.857142857 |
| HLA-C*01 | 0.446813725 | 0.389828431 | 0.596568627 | 0.364460784 |
| HLA-C*02 | 0.28265044 | 0.312170143 | 0.418420433 | 0.282143165 |
| HLA-C*03 | 0.57340264 | 0.609736239 | 0.574869991 | 0.579534945 |
| HLA-C*04 | 0.173900373 | 0.162178482 | 0.252728279 | 0.181510324 |
| HLA-C*05 | 0.295979682 | 0.221736399 | 0.25984167 | 0.214160192 |
| HLA-C*06 | 0.226735061 | 0.29070006 | 0.320339452 | 0.311414181 |
| HLA-C*07 | 0.134165761 | 0.147611396 | 0.216631282 | 0.175518599 |
| HLA-C*08 | 0.333333333 | 0.333333333 | 0.5 | 0.5 |
| HLA-C*12 | 0.258492689 | 0.297358643 | 0.409945994 | 0.303525493 |
| HLA-C*14 | 0.25 | 0.225 | 0.766666667 | 0.683333333 |
| HLA-C*15 | 0 | 0.5 | 0.5 | 0 |
| HLA-C*16 | 0.062694013 | 0.049390244 | 0.102839721 | 0.130899588 |

**Supplementary Table 3. Means of PPVs for Different Alleles in TeSet-1.** The means were calculated for all MHC-1 molecules sharing the same gene and allelic group, e.g. HLA-A*02.
