# Supplementary material for: NeoMUST: an accurate and efficient multi-task learning model for neoantigen presentation
Source: Life Sci Alliance. 2024 Jan 30;7(4):e202302255. doi: 10.26508/lsa.202302255 (PMC10828515; doi:10.26508/lsa.202302255)
Supplement: Supplementary file 5 [file LSA-2023-02255_TableS4.docx]

# 4 Supplementary Table 4

| HLA | NetMHCpan4.0 EL | MHCflurry2.0 BA | MHCflurry2.0 PS | NeoMUST NP |
| --- | --- | --- | --- | --- |
| HLA-A*01 | 0.439606618 | 0.418024484 | 0.388210442 | 0.415299271 |
| HLA-A*02 | 0.321206398 | 0.236361574 | 0.373827489 | 0.321230278 |
| HLA-A*03 | 0.25449303 | 0.229065898 | 0.256689491 | 0.221296976 |
| HLA-A*11 | 0.226140761 | 0.197698352 | 0.241714385 | 0.21355142 |
| HLA-A*23 | 0.669413202 | 0.646355052 | 0.748265651 | 0.693984669 |
| HLA-A*24 | 0.498012977 | 0.517562313 | 0.594811944 | 0.518267914 |
| HLA-A*25 | 0.766299895 | 0.805319406 | 0.830739222 | 0.760602038 |
| HLA-A*26 | 0.807593834 | 0.82339215 | 0.860405842 | 0.816247218 |
| HLA-A*29 | 0.23017671 | 0.19676642 | 0.218146373 | 0.217755002 |
| HLA-A*30 | 0.367805213 | 0.366496281 | 0.420968029 | 0.433669716 |
| HLA-A*31 | 0.287491853 | 0.255575003 | 0.34056738 | 0.322199758 |
| HLA-A*32 | 0.331066493 | 0.351519933 | 0.429260253 | 0.342682766 |
| HLA-A*68 | 0.494903054 | 0.496397513 | 0.587165796 | 0.584407597 |
| HLA-B*07 | 0.392045147 | 0.373661313 | 0.371885221 | 0.357015058 |
| HLA-B*08 | 0.511414595 | 0.548863492 | 0.479012574 | 0.497960185 |
| HLA-B*13 | 0.436893665 | 0.501293285 | 0.553849853 | 0.509200611 |
| HLA-B*14 | 0.431068561 | 0.40572232 | 0.586779072 | 0.525466731 |
| HLA-B*15 | 0.660502139 | 0.637165426 | 0.709358129 | 0.699082076 |
| HLA-B*18 | 0.35874998 | 0.415861765 | 0.401781642 | 0.360544273 |
| HLA-B*27 | 0.079258286 | 0.078966255 | 0.103382235 | 0.017354305 |
| HLA-B*35 | 0.615007191 | 0.605533034 | 0.648762707 | 0.601289455 |
| HLA-B*37 | 0.387110301 | 0.429726736 | 0.488625997 | 0.557133285 |
| HLA-B*38 | 0.626247639 | 0.711359679 | 0.826242012 | 0.794927332 |
| HLA-B*39 | 0.599463186 | 0.747718267 | 0.698797011 | 0.741047034 |
| HLA-B*40 | 0.36265621 | 0.40239934 | 0.401967317 | 0.413777354 |
| HLA-B*41 | 0.299456295 | 0.48533971 | 0.458085603 | 0.763823612 |
| HLA-B*44 | 0.395889829 | 0.369820683 | 0.340511492 | 0.409984112 |
| HLA-B*45 | 0.621364744 | 0.711993486 | 0.62447736 | 0.748667151 |
| HLA-B*47 | 0.941734761 | 0.944036176 | 0.926066374 | 0.924614699 |
| HLA-B*50 | 0.590968232 | 0.631415643 | 0.708836814 | 0.648550445 |
| HLA-B*51 | 0.212953096 | 0.320691634 | 0.289377289 | 0.357327817 |
| HLA-B*55 | 0.608777068 | 0.659878674 | 0.728783914 | 0.627476928 |
| HLA-B*56 | 0.395604873 | 0.504907321 | 0.513917017 | 0.44646429 |
| HLA-B*57 | 0.446658261 | 0.394494685 | 0.430853116 | 0.401341654 |
| HLA-B*58 | 0.350834227 | 0.310850372 | 0.481880116 | 0.283204087 |
| HLA-B*73 | 0.315028845 | 0.563925429 | 0.554396634 | 0.681379871 |
| HLA-C*01 | 0.250361994 | 0.30867281 | 0.480632499 | 0.286932248 |
| HLA-C*02 | 0.152979893 | 0.183396122 | 0.293200868 | 0.179755841 |
| HLA-C*03 | 0.379220031 | 0.415141563 | 0.413342793 | 0.399616978 |
| HLA-C*04 | 0.133272494 | 0.086219163 | 0.213090036 | 0.129190023 |
| HLA-C*05 | 0.194167307 | 0.149846594 | 0.186859955 | 0.149596332 |
| HLA-C*06 | 0.240377738 | 0.326154963 | 0.360121354 | 0.271796661 |
| HLA-C*07 | 0.138626202 | 0.133284388 | 0.213742825 | 0.132105253 |
| HLA-C*08 | 0.275531533 | 0.260106501 | 0.350841629 | 0.304213687 |
| HLA-C*12 | 0.168378669 | 0.200907789 | 0.301118708 | 0.204327538 |
| HLA-C*14 | 0.32585358 | 0.322048189 | 0.593142824 | 0.495756879 |
| HLA-C*15 | 0.295833333 | 0.730555556 | 0.756944444 | 0.381944444 |
| HLA-C*16 | 0.066091736 | 0.059723057 | 0.068568578 | 0.082027549 |

**Supplementary Table 4. Means of AUC-PRs for Different Alleles in TeSet-1.** The means were calculated for all MHC-1 molecules sharing the same gene and allelic group, e.g. HLA-A*02.
