# Supplementary material for: NeoMUST: an accurate and efficient multi-task learning model for neoantigen presentation
Source: Life Sci Alliance. 2024 Jan 30;7(4):e202302255. doi: 10.26508/lsa.202302255 (PMC10828515; doi:10.26508/lsa.202302255)
Supplement: Supplementary file 7 [file LSA-2023-02255_TableS5.docx]

# 5 Supplementary Table 5

| HLA | seq_count | NetMHCpan4.0 EL | MHCflurry2.0 BA | NeoMUST NP |
| --- | --- | --- | --- | --- |
| HLA-A*01 | 8200 | 0.963664005 | 0.965247358 | 0.973522404 |
| HLA-A*02 | 1370400 | 0.970157377 | 0.971746979 | 0.971690108 |
| HLA-A*03 | 11200 | 0.935642297 | 0.937355056 | 0.949295248 |
| HLA-A*11 | 614700 | 0.971620795 | 0.971244384 | 0.975808893 |
| HLA-A*23 | 226500 | 0.945954687 | 0.943034195 | 0.958196082 |
| HLA-A*24 | 154600 | 0.978100948 | 0.981963566 | 0.984663958 |
| HLA-A*25 | 52300 | 0.963268405 | 0.971512965 | 0.969125058 |
| HLA-A*26 | 123800 | 0.977321702 | 0.976424942 | 0.978877299 |
| HLA-A*29 | 8900 | 0.859634089 | 0.875108872 | 0.864383642 |
| HLA-A*30 | 344800 | 0.958415866 | 0.953941296 | 0.964268848 |
| HLA-A*31 | 10800 | 0.939338948 | 0.946273434 | 0.966351183 |
| HLA-A*32 | 219200 | 0.977788899 | 0.978795574 | 0.982264784 |
| HLA-A*33 | 523900 | 0.985801451 | 0.978839484 | 0.980326728 |
| HLA-A*34 | 575500 | 0.974730035 | 0.974756233 | 0.97988264 |
| HLA-A*36 | 195300 | 0.942997059 | 0.94835582 | 0.942798004 |
| HLA-A*66 | 198300 | 0.975044336 | 0.977792053 | 0.980344419 |
| HLA-A*68 | 111600 | 0.853881981 | 0.872838295 | 0.918827167 |
| HLA-A*74 | 245800 | 0.982629887 | 0.982183067 | 0.985281319 |
| HLA-B*07 | 192200 | 0.951515649 | 0.953101576 | 0.952098034 |
| HLA-B*08 | 25500 | 0.984636523 | 0.986310626 | 0.983002575 |
| HLA-B*13 | 629500 | 0.968325339 | 0.943998773 | 0.974459313 |
| HLA-B*14 | 126900 | 0.971454794 | 0.97524974 | 0.980896636 |
| HLA-B*15 | 774200 | 0.981149012 | 0.980905983 | 0.979763851 |
| HLA-B*18 | 107500 | 0.978928941 | 0.980013199 | 0.980418371 |
| HLA-B*27 | 15500 | 0.885668338 | 0.922352768 | 0.893575926 |
| HLA-B*35 | 323900 | 0.91672821 | 0.940339294 | 0.97556035 |
| HLA-B*37 | 154200 | 0.935875801 | 0.93169238 | 0.944236882 |
| HLA-B*38 | 578400 | 0.985927874 | 0.984954761 | 0.983956518 |
| HLA-B*40 | 478500 | 0.976039057 | 0.979064702 | 0.979315309 |
| HLA-B*42 | 339200 | 0.988068287 | 0.986800864 | 0.975037036 |
| HLA-B*44 | 12100 | 0.952407825 | 0.964308605 | 0.978288695 |
| HLA-B*45 | 124500 | 0.952311351 | 0.958419026 | 0.960779423 |
| HLA-B*46 | 61600 | 0.959457124 | 0.966714816 | 0.972732269 |
| HLA-B*49 | 369700 | 0.977397581 | 0.975497596 | 0.979298317 |
| HLA-B*50 | 53600 | 0.973374116 | 0.976705228 | 0.975676672 |
| HLA-B*51 | 19100 | 0.841183275 | 0.859456382 | 0.942151152 |
| HLA-B*52 | 207300 | 0.965913819 | 0.963316755 | 0.97432428 |
| HLA-B*53 | 221000 | 0.989010228 | 0.990180903 | 0.987333931 |
| HLA-B*54 | 26300 | 0.872018775 | 0.89556935 | 0.951828642 |
| HLA-B*55 | 286200 | 0.983458741 | 0.986018875 | 0.986135274 |
| HLA-B*56 | 129500 | 0.954359733 | 0.961490717 | 0.957394472 |
| HLA-B*57 | 136300 | 0.859919382 | 0.867354047 | 0.918897175 |
| HLA-B*58 | 195800 | 0.967361445 | 0.954766975 | 0.961748476 |
| HLA-C*01 | 73100 | 0.974558105 | 0.984596537 | 0.984143272 |
| HLA-C*02 | 46800 | 0.870230632 | 0.938409499 | 0.83431563 |
| HLA-C*03 | 523100 | 0.917031209 | 0.924554762 | 0.924227893 |
| HLA-C*04 | 202800 | 0.972900826 | 0.976528324 | 0.977487859 |
| HLA-C*05 | 51500 | 0.960911043 | 0.965338102 | 0.974599287 |
| HLA-C*06 | 131000 | 0.877396841 | 0.895740528 | 0.90783957 |
| HLA-C*07 | 219600 | 0.943025613 | 0.949559045 | 0.875282398 |
| HLA-C*08 | 366100 | 0.974089281 | 0.979065113 | 0.964068948 |
| HLA-C*12 | 163200 | 0.960685554 | 0.969886179 | 0.978818818 |
| HLA-C*14 | 379500 | 0.982568711 | 0.984505822 | 0.98638622 |
| HLA-C*15 | 240100 | 0.971864021 | 0.972873722 | 0.976308392 |
| HLA-C*16 | 212100 | 0.914774445 | 0.919748492 | 0.887459441 |
| HLA-C*17 | 78300 | 0.955468385 | 0.968037267 | 0.965887596 |

**Supplementary Table 5. Means of AUC-ROCs for Different Alleles in TeSet-2.** The means were calculated for all MHC-1 molecules sharing the same gene and allelic group, e.g. HLA-A*02.
