# Supplementary material for: NeoMUST: an accurate and efficient multi-task learning model for neoantigen presentation
Source: Life Sci Alliance. 2024 Jan 30;7(4):e202302255. doi: 10.26508/lsa.202302255 (PMC10828515; doi:10.26508/lsa.202302255)
Supplement: Supplementary file 8 [file LSA-2023-02255_TableS6.docx]

# 6 Supplementary Table 6

| HLA | seq_count | NetMHCpan4.0 EL | MHCflurry2.0 BA | NeoMUST NP |
| --- | --- | --- | --- | --- |
| HLA-A*01 | 8200 | 0.695121951 | 0.719512195 | 0.743902439 |
| HLA-A*02 | 1370400 | 0.632716193 | 0.595073548 | 0.609381496 |
| HLA-A*03 | 11200 | 0.375 | 0.410714286 | 0.392857143 |
| HLA-A*11 | 614700 | 0.612094515 | 0.600326082 | 0.607032339 |
| HLA-A*23 | 226500 | 0.695364238 | 0.72406181 | 0.727152318 |
| HLA-A*24 | 154600 | 0.743321681 | 0.745085972 | 0.742626906 |
| HLA-A*25 | 52300 | 0.583173996 | 0.694072658 | 0.707456979 |
| HLA-A*26 | 123800 | 0.71082391 | 0.708400646 | 0.751211632 |
| HLA-A*29 | 8900 | 0.393258427 | 0.415730337 | 0.382022472 |
| HLA-A*30 | 344800 | 0.546338931 | 0.497435558 | 0.592517322 |
| HLA-A*31 | 10800 | 0.361111111 | 0.361111111 | 0.361111111 |
| HLA-A*32 | 219200 | 0.638433386 | 0.668300586 | 0.733116348 |
| HLA-A*33 | 523900 | 0.631333908 | 0.541377532 | 0.458105826 |
| HLA-A*34 | 575500 | 0.582379912 | 0.541661117 | 0.579813236 |
| HLA-A*36 | 195300 | 0.693804403 | 0.688684076 | 0.618535586 |
| HLA-A*66 | 198300 | 0.627836611 | 0.673726677 | 0.708522441 |
| HLA-A*68 | 111600 | 0.451904899 | 0.46626954 | 0.463494017 |
| HLA-A*74 | 245800 | 0.619609439 | 0.624898291 | 0.589096827 |
| HLA-B*07 | 192200 | 0.605712597 | 0.582739811 | 0.554132949 |
| HLA-B*08 | 25500 | 0.615686275 | 0.678431373 | 0.596078431 |
| HLA-B*13 | 629500 | 0.426101039 | 0.336639434 | 0.540223677 |
| HLA-B*14 | 126900 | 0.595744681 | 0.626477541 | 0.699763593 |
| HLA-B*15 | 774200 | 0.686443671 | 0.655341219 | 0.652866929 |
| HLA-B*18 | 107500 | 0.626976744 | 0.707906977 | 0.717209302 |
| HLA-B*27 | 15500 | 0.451612903 | 0.451612903 | 0.2 |
| HLA-B*35 | 323900 | 0.64862032 | 0.64891066 | 0.623477133 |
| HLA-B*37 | 154200 | 0.483138781 | 0.409857328 | 0.614137484 |
| HLA-B*38 | 578400 | 0.683768248 | 0.699406508 | 0.715740508 |
| HLA-B*40 | 478500 | 0.627734113 | 0.664775662 | 0.701815534 |
| HLA-B*42 | 339200 | 0.686320755 | 0.673643868 | 0.476709906 |
| HLA-B*44 | 12100 | 0.71969697 | 0.760606061 | 0.709090909 |
| HLA-B*45 | 124500 | 0.650963298 | 0.699218522 | 0.726478455 |
| HLA-B*46 | 61600 | 0.517857143 | 0.607142857 | 0.607142857 |
| HLA-B*49 | 369700 | 0.731674331 | 0.703272924 | 0.707600757 |
| HLA-B*50 | 53600 | 0.626865672 | 0.61380597 | 0.61380597 |
| HLA-B*51 | 19100 | 0.528795812 | 0.523560209 | 0.539267016 |
| HLA-B*52 | 207300 | 0.4911686 | 0.509268983 | 0.597892211 |
| HLA-B*53 | 221000 | 0.769683258 | 0.746606335 | 0.67239819 |
| HLA-B*54 | 26300 | 0.539923954 | 0.604562738 | 0.593155894 |
| HLA-B*55 | 286200 | 0.636710769 | 0.653557616 | 0.656257746 |
| HLA-B*56 | 129500 | 0.573745174 | 0.627799228 | 0.644015444 |
| HLA-B*57 | 136300 | 0.517270864 | 0.53407261 | 0.516359245 |
| HLA-B*58 | 195800 | 0.617256171 | 0.564676213 | 0.565929825 |
| HLA-C*01 | 73100 | 0.477428181 | 0.630642955 | 0.647058824 |
| HLA-C*02 | 46800 | 0.414529915 | 0.510683761 | 0.536324786 |
| HLA-C*03 | 523100 | 0.537037859 | 0.562744856 | 0.55981337 |
| HLA-C*04 | 202800 | 0.675229144 | 0.649809699 | 0.666247607 |
| HLA-C*05 | 51500 | 0.615533981 | 0.739805825 | 0.737864078 |
| HLA-C*06 | 131000 | 0.426812865 | 0.528684211 | 0.511637427 |
| HLA-C*07 | 219600 | 0.458496994 | 0.432679543 | 0.376471139 |
| HLA-C*08 | 366100 | 0.612236036 | 0.65269928 | 0.587623523 |
| HLA-C*12 | 163200 | 0.515396341 | 0.553506098 | 0.584756098 |
| HLA-C*14 | 379500 | 0.625955926 | 0.679095895 | 0.706927355 |
| HLA-C*15 | 240100 | 0.551020408 | 0.513119534 | 0.58850479 |
| HLA-C*16 | 212100 | 0.361150401 | 0.378595002 | 0.417256011 |
| HLA-C*17 | 78300 | 0.455938697 | 0.563218391 | 0.624521073 |

**Supplementary Table 6. Means of PPV for Different Alleles in TeSet-2.** The means were calculated for all MHC-1 molecules sharing the same gene and allelic group, e.g. HLA-A*02.
