# Supplementary material for: NeoMUST: an accurate and efficient multi-task learning model for neoantigen presentation
Source: Life Sci Alliance. 2024 Jan 30;7(4):e202302255. doi: 10.26508/lsa.202302255 (PMC10828515; doi:10.26508/lsa.202302255)
Supplement: Supplementary file 9 [file LSA-2023-02255_TableS7.docx]

# 7 Supplementary Table 7

| HLA | seq_count | NetMHCpan4.0 EL | MHCflurry2.0 BA | NeoMUST NP |
| --- | --- | --- | --- | --- |
| HLA-A*01 | 8200 | 0.745255583 | 0.743898537 | 0.743902439 |
| HLA-A*02 | 1370400 | 0.643328167 | 0.581117477 | 0.609381496 |
| HLA-A*03 | 11200 | 0.705298084 | 0.699859675 | 0.607142857 |
| HLA-A*11 | 614700 | 0.634581462 | 0.589856785 | 0.607032339 |
| HLA-A*23 | 226500 | 0.70845419 | 0.71797791 | 0.727152318 |
| HLA-A*24 | 154600 | 0.741316947 | 0.766373438 | 0.742626906 |
| HLA-A*25 | 52300 | 0.552180253 | 0.688662323 | 0.707456979 |
| HLA-A*26 | 123800 | 0.764058844 | 0.747141283 | 0.751211632 |
| HLA-A*29 | 8900 | 0.679033289 | 0.646432862 | 0.617977528 |
| HLA-A*30 | 344800 | 0.525729124 | 0.531509528 | 0.592517322 |
| HLA-A*31 | 10800 | 0.740064279 | 0.715877657 | 0.638888889 |
| HLA-A*32 | 219200 | 0.679630996 | 0.691951744 | 0.733116348 |
| HLA-A*33 | 523900 | 0.671576607 | 0.513461571 | 0.541894174 |
| HLA-A*34 | 575500 | 0.588460357 | 0.530517483 | 0.579813236 |
| HLA-A*36 | 195300 | 0.694092518 | 0.690676919 | 0.618535586 |
| HLA-A*66 | 198300 | 0.620792805 | 0.711376386 | 0.708522441 |
| HLA-A*68 | 111600 | 0.601503704 | 0.618111917 | 0.536505983 |
| HLA-A*74 | 245800 | 0.660662887 | 0.651085735 | 0.589096827 |
| HLA-B*07 | 192200 | 0.619227648 | 0.570865492 | 0.554132949 |
| HLA-B*08 | 25500 | 0.635557781 | 0.732125496 | 0.596078431 |
| HLA-B*13 | 629500 | 0.614483825 | 0.717197384 | 0.540223677 |
| HLA-B*14 | 126900 | 0.620373382 | 0.645462587 | 0.699763593 |
| HLA-B*15 | 774200 | 0.726564782 | 0.679542685 | 0.652866929 |
| HLA-B*18 | 107500 | 0.640245296 | 0.735659446 | 0.717209302 |
| HLA-B*27 | 15500 | 0.598933866 | 0.60599733 | 0.8 |
| HLA-B*35 | 323900 | 0.671286187 | 0.672520247 | 0.623477133 |
| HLA-B*37 | 154200 | 0.524317371 | 0.639063108 | 0.614137484 |
| HLA-B*38 | 578400 | 0.735945812 | 0.74843307 | 0.715740508 |
| HLA-B*40 | 478500 | 0.638266151 | 0.67459974 | 0.701815534 |
| HLA-B*42 | 339200 | 0.735410322 | 0.700474448 | 0.523290094 |
| HLA-B*44 | 12100 | 0.695686731 | 0.656432426 | 0.709090909 |
| HLA-B*45 | 124500 | 0.689878733 | 0.715228811 | 0.726478455 |
| HLA-B*46 | 61600 | 0.511842964 | 0.632898212 | 0.607142857 |
| HLA-B*49 | 369700 | 0.774869275 | 0.734518057 | 0.707600757 |
| HLA-B*50 | 53600 | 0.646753415 | 0.625414996 | 0.61380597 |
| HLA-B*51 | 19100 | 0.525873314 | 0.535924972 | 0.539267016 |
| HLA-B*52 | 207300 | 0.517836739 | 0.507524632 | 0.597892211 |
| HLA-B*53 | 221000 | 0.823072828 | 0.798855318 | 0.67239819 |
| HLA-B*54 | 26300 | 0.535976557 | 0.558527486 | 0.593155894 |
| HLA-B*55 | 286200 | 0.674986081 | 0.69261106 | 0.656257746 |
| HLA-B*56 | 129500 | 0.562431836 | 0.627857586 | 0.644015444 |
| HLA-B*57 | 136300 | 0.501619064 | 0.50268674 | 0.516359245 |
| HLA-B*58 | 195800 | 0.605085102 | 0.528419847 | 0.565929825 |
| HLA-C*01 | 73100 | 0.542384502 | 0.667686613 | 0.647058824 |
| HLA-C*02 | 46800 | 0.650072994 | 0.548477686 | 0.536324786 |
| HLA-C*03 | 523100 | 0.543198386 | 0.567801321 | 0.55981337 |
| HLA-C*04 | 202800 | 0.711519031 | 0.685840105 | 0.666247607 |
| HLA-C*05 | 51500 | 0.647403948 | 0.768632091 | 0.737864078 |
| HLA-C*06 | 131000 | 0.602999081 | 0.505608225 | 0.511637427 |
| HLA-C*07 | 219600 | 0.568623664 | 0.582672447 | 0.623528861 |
| HLA-C*08 | 366100 | 0.63543655 | 0.680335505 | 0.587623523 |
| HLA-C*12 | 163200 | 0.523101309 | 0.574568849 | 0.584756098 |
| HLA-C*14 | 379500 | 0.656521183 | 0.702514081 | 0.706927355 |
| HLA-C*15 | 240100 | 0.559739598 | 0.513586286 | 0.58850479 |
| HLA-C*16 | 212100 | 0.696140717 | 0.668220807 | 0.582743989 |
| HLA-C*17 | 78300 | 0.610610486 | 0.58227334 | 0.624521073 |

**Supplementary Table 7. Means of AUC-PRs for Different Alleles in TeSet-2.** The means were calculated for all MHC-1 molecules sharing the same gene and allelic group, e.g. HLA-A*02.
