# Supplementary material for: NeoMUST: an accurate and efficient multi-task learning model for neoantigen presentation
Source: Life Sci Alliance. 2024 Jan 30;7(4):e202302255. doi: 10.26508/lsa.202302255 (PMC10828515; doi:10.26508/lsa.202302255)
Supplement: Supplementary file 12 [file LSA-2023-02255_TableS8.docx]

### Supplementary Table 8

| Patient | Peptide Mut | Peptide Wild | Pep Length | HLA | Agretopicity | Hit | NetMHCpan4.1 | MixMHCpred2.2 | MHCflurry2.0 BA | MHCflurry2.0 PS | NeoMUST |
| --- | --- | --- | --- | --- | --- | --- | --- | --- | --- | --- | --- |
| 1 | TELERFLEY | TELERSLEY | 9 | HLA-B*44:02 | 0.719 | 0 | 0.015 | 0.036 | 39.889 | 0.983 | 0.003 |
| 1 | TLFHTFYEL | TLFHTFYDL | 9 | HLA-A*02:01 | 0.179 | 0 | 0.112 | 0.061 | 12.138 | 0.977 | 0.004 |
| 1 | KFGDLTNNF | KFGDLTDNF | 9 | HLA-A*24:02 | 0.592 | 0 | 0.044 | 0.033 | 43.257 | 0.872 | 0.008 |
| 1 | RFLEYLPLRF | RSLEYLPLRF | 10 | HLA-A*24:02 | 0.067 | 1 | 0.104 | 0.066 | 59.717 | 0.951 | 0.011 |
| 1 | FLFQDSKKI | FFFQDSKKI | 9 | HLA-A*02:01 | 0.005 | 0 | 0.166 | 0.266 | 23.076 | 0.912 | 0.018 |
| 1 | GIPENSFNV | GIPGNSFNV | 9 | HLA-A*02:01 | 0.980 | 0 | 0.366 | 0.356 | 56.915 | 0.665 | 0.020 |
| 1 | ALFASRPRF | VLFASRPRF | 9 | HLA-B*15:01 | 0.838 | 0 | 0.141 | 0.338 | 79.981 | 0.887 | 0.028 |
| 1 | KLKFVTLVF | ELKFVTLVF | 9 | HLA-B*15:01 | 0.155 | 1 | 0.049 | 0.051 | 44.333 | 0.797 | 0.028 |
| 1 | LFHTFYELL | LFHTFYDLL | 9 | HLA-A*24:02 | 0.711 | 0 | 0.636 | 0.191 | 67.983 | 0.647 | 0.029 |
| 1 | KLFESKAELA | QLFESKAELA | 10 | HLA-A*02:01 | 0.207 | 0 | 0.537 | 0.248 | 62.788 | 0.822 | 0.032 |
| 1 | SFSSAPMPQI | SFSSASMPQI | 10 | HLA-A*24:02 | 0.880 | 0 | 1.929 | 2.040 | 1432.554 | 0.066 | 0.046 |
| 1 | VLAKKLKFV | VLAKELKFV | 9 | HLA-A*02:01 | 1.855 | 0 | 0.245 | 0.085 | 31.253 | 0.922 | 0.055 |
| 1 | LLHTELERF | LLHTELERS | 9 | HLA-B*15:01 | 0.010 | 0 | 0.316 | 0.238 | 111.500 | 0.730 | 0.056 |
| 1 | VQKVASKIPF | VQKVASKIPS | 10 | HLA-B*15:01 | 0.007 | 0 | 0.491 | 0.258 | 109.653 | 0.573 | 0.065 |
| 1 | KLKFVTLVF | ELKFVTLVF | 9 | HLA-A*24:02 | 0.125 | 1 | 0.978 | 0.623 | 818.412 | 0.188 | 0.078 |
| 1 | LFHTFYELLI | LFHTFYDLLI | 10 | HLA-A*24:02 | 0.831 | 0 | 7.586 | 4.788 | 4795.425 | 0.041 | 0.102 |
| 1 | NSKKKWFLF | NSKKKWFFF | 9 | HLA-A*24:02 | 0.466 | 0 | 1.098 | 0.510 | 765.949 | 0.286 | 0.109 |
| 1 | VSVGDFSQEF | VSVGDSSQEF | 10 | HLA-B*15:01 | 0.251 | 0 | 1.952 | 1.111 | 1354.846 | 0.113 | 0.153 |
| 1 | TTLFHTFYEL | TTLFHTFYDL | 10 | HLA-A*24:02 | 0.363 | 0 | 8.639 | 14.171 | 16847.016 | 0.013 | 0.158 |
| 1 | TLFHTFYELL | TLFHTFYDLL | 10 | HLA-A*02:01 | 0.572 | 0 | 1.950 | 1.571 | 151.827 | 0.397 | 0.169 |

Supplementary Table 8 Continued

| Patient | Peptide Mut | Peptide Wild | Pep Length | HLA | Agretopicity | Hit | NetMHCpan4.1 | MixMHCpred2.2 | MHCflurry2.0 BA | MHCflurry2.0 PS | NeoMUST |
| --- | --- | --- | --- | --- | --- | --- | --- | --- | --- | --- | --- |
| 1 | LLHTELERFL | LLHTELERSL | 10 | HLA-A*02:01 | 0.507 | 0 | 2.229 | 3.159 | 2317.742 | 0.056 | 0.223 |
| 1 | YNSFSSAPM | YNSFSSASM | 9 | HLA-B*15:01 | 0.207 | 0 | 11.655 | 8.342 | 4761.856 | 0.020 | 0.277 |
| 1 | TTLFHTFYEL | TTLFHTFYDL | 10 | HLA-A*02:01 | 0.218 | 0 | 2.216 | 5.977 | 4959.232 | 0.043 | 0.353 |
| 2 | HPAPPAPPPA | LPAPPAPPPA | 10 | HLA-B*56:01 | 0.500 | 0 | 0.007 | 0.001 | 32.079 | 0.751 | 0.001 |
| 2 | FLDREQRESY | SLDREQRESY | 10 | HLA-A*01:01 | 0.333 | 0 | 0.054 | 0.003 | 49.503 | 0.948 | 0.009 |
| 2 | YTWPSGNIY | YTWPSGNTY | 9 | HLA-A*01:01 | 5.000 | 0 | 0.055 | 0.063 | 53.910 | 0.896 | 0.010 |
| 2 | FPKKIQMLA | FPKEIQMLA | 9 | HLA-B*56:01 | 1.263 | 1 | 0.006 | 0.659 | 37.213 | 0.781 | 0.026 |
| 2 | LADFRLARLY | LADFGLARLY | 10 | HLA-A*01:01 | 0.736 | 0 | 0.186 | 0.251 | 74.867 | 0.699 | 0.039 |
| 2 | FPGNQWNPV | FPGNRWNPV | 9 | HLA-B*56:01 | 1.106 | 0 | 0.377 | 0.703 | 68.588 | 0.562 | 0.042 |
| 2 | ITDAHELGVA | ITAAHELGVA | 10 | HLA-A*01:01 | 5.601 | 0 | 2.105 | 2.248 | 1600.769 | 0.155 | 0.076 |
| 3 | RTAPRPGSQK | RTAPRPGSQE | 10 | HLA-A*03:01 | 0.737 | 0 | 0.008 | 0.001 | 31.813 | 0.762 | 0.000 |
| 3 | RIYTGEKPFK | RIHTGEKPFK | 10 | HLA-A*03:01 | 0.002 | 0 | 0.018 | 0.019 | 29.038 | 0.986 | 0.002 |
| 3 | ALSPDGSIRK | APSPDGSIRK | 10 | HLA-A*03:01 | 3.511 | 0 | 0.054 | 0.157 | 50.846 | 0.803 | 0.002 |
| 3 | TLLSQVNKV | TPLSQVNKV | 9 | HLA-A*02:01 | 0.999 | 0 | 0.029 | 0.005 | 21.511 | 0.987 | 0.006 |
| 3 | ALQSQSISL | VLQSQSISL | 9 | HLA-A*02:01 | 0.451 | 0 | 0.104 | 0.012 | 60.397 | 0.696 | 0.017 |
| 3 | LLFAPEYGPK | LLSAPEYGPK | 10 | HLA-A*03:01 | 1.399 | 0 | 0.727 | 0.332 | 66.032 | 0.841 | 0.025 |
| 3 | KIIAYQPYGK | EIIAYQPYGK | 10 | HLA-A*03:01 | 0.323 | 0 | 0.224 | 0.285 | 33.287 | 0.914 | 0.026 |
| 3 | RLMLRKVALK | RLMLRRVALK | 10 | HLA-A*03:01 | 0.200 | 0 | 0.161 | 0.102 | 32.446 | 0.809 | 0.027 |
| 3 | LRAAFFGKCF | LRAASFGKCF | 10 | HLA-B*27:05 | 0.006 | 1 | 2.327 | 0.727 | 178.925 | 0.382 | 0.029 |
| 3 | YLLFQNTDL | YLLFQDTDL | 9 | HLA-A*02:01 | 0.924 | 0 | 0.397 | 0.558 | 29.860 | 0.845 | 0.038 |
| 3 | SLLRAAFFGK | SLLRAASFGK | 10 | HLA-A*03:01 | 0.465 | 0 | 0.512 | 0.450 | 36.010 | 0.801 | 0.040 |

Supplementary Table 8 Continued

| Patient | Peptide Mut | Peptide Wild | Pep Length | HLA | Agretopicity | Hit | NetMHCpan4.1 | MixMHCpred2.2 | MHCflurry2.0 BA | MHCflurry2.0 PS | NeoMUST |
| --- | --- | --- | --- | --- | --- | --- | --- | --- | --- | --- | --- |
| 3 | LRFNLIANQH | LRFNLTANQH | 10 | HLA-B*27:05 | 2.794 | 0 | 0.873 | 0.416 | 141.659 | 0.464 | 0.040 |
| 3 | SLPSNVLSSL | SLPSNVLPSL | 10 | HLA-A*02:01 | 1.250 | 0 | 0.934 | 0.215 | 54.842 | 0.838 | 0.040 |
| 3 | SLHKGSLYPR | PLHKGSLYPR | 10 | HLA-A*03:01 | 1.120 | 0 | 0.609 | 1.284 | 204.051 | 0.441 | 0.053 |
| 3 | RLMLRKVAL | RLMLRRVAL | 9 | HLA-A*02:01 | 0.073 | 0 | 0.659 | 0.467 | 257.605 | 0.728 | 0.067 |
| 3 | VRTLLSQVNK | VRTPLSQVNK | 10 | HLA-B*27:05 | 0.838 | 1 | 0.829 | 0.640 | 151.285 | 0.518 | 0.094 |
| 3 | KLNFRLFVIR | KLNSRLFVIR | 10 | HLA-A*03:01 | 0.059 | 0 | 1.947 | 2.358 | 791.820 | 0.125 | 0.103 |
| 3 | QRLMLRKVAL | QRLMLRRVAL | 10 | HLA-B*27:05 | 0.050 | 0 | 0.847 | 0.372 | 98.181 | 0.641 | 0.149 |
| 3 | KLNFRLFVI | KLNSRLFVI | 9 | HLA-A*02:01 | 0.305 | 0 | 1.365 | 0.935 | 41.386 | 0.838 | 0.169 |
| 3 | FLLTDYALS | FLLTDYAPS | 9 | HLA-A*02:01 | 0.024 | 0 | 3.337 | 5.361 | 906.208 | 0.093 | 0.230 |
| 3 | SLQRLMLRKV | SLQRLMLRRV | 10 | HLA-A*02:01 | 0.804 | 0 | 3.677 | 1.195 | 1288.627 | 0.086 | 0.288 |
| 3 | WRNILLLSLH | WRNILLLPLH | 10 | HLA-B*27:05 | 1.893 | 1 | 2.113 | 1.397 | 1841.455 | 0.050 | 0.369 |
| 3 | RKLNFRLFVI | RKLNSRLFVI | 10 | HLA-B*27:05 | 1.156 | 0 | 5.111 | 6.073 | 2163.910 | 0.192 | 0.526 |
| 5 | FPQGLPNEY | FPQGLPDEY | 9 | HLA-B*35:01 | 0.059 | 1 | 0.003 | 0.005 | 24.768 | 0.958 | 0.000 |
| 5 | TIIDNIKEM | TIIDNIEEM | 9 | HLA-A*66:01 | 0.195 | 0 | 0.085 | 0.788 | 361.329 | 0.814 | 0.005 |
| 5 | MAGPKGFQY | MAGPEGFQY | 9 | HLA-B*35:01 | 2.109 | 0 | 0.058 | 0.113 | 28.504 | 0.957 | 0.008 |
| 5 | RYGIRGFSTI | RYGIRGFPTI | 10 | HLA-A*23:01 | 0.175 | 0 | 0.420 | 1.442 | 399.749 | 0.307 | 0.014 |
| 5 | MPFTTVSELM | MPFTTVSELV | 10 | HLA-B*35:01 | 0.372 | 0 | 0.233 | 0.086 | 51.887 | 0.752 | 0.015 |
| 5 | LFSPGAANLF | LSSPGAANLF | 10 | HLA-A*23:01 | 0.031 | 0 | 0.271 | 0.311 | 56.271 | 0.809 | 0.017 |
| 5 | EYPVIFKSI | EYPVISKSI | 9 | HLA-A*23:01 | 0.415 | 0 | 0.188 | 0.058 | 96.270 | 0.908 | 0.022 |
| 5 | SELMKVSAM | SELVKVSAM | 9 | HLA-B*41:02 | 0.357 | 0 | 0.017 | 0.093 | 38.679 | 0.972 | 0.023 |
| 5 | RFANPRDSF | LFANPRDSF | 9 | HLA-A*23:01 | 0.686 | 0 | 0.264 | 0.286 | 89.822 | 0.773 | 0.023 |

Supplementary Table 8 Continued

| Patient | Peptide Mut | Peptide Wild | Pep Length | HLA | Agretopicity | Hit | NetMHCpan4.1 | MixMHCpred2.2 | MHCflurry2.0 BA | MHCflurry2.0 PS | NeoMUST |
| --- | --- | --- | --- | --- | --- | --- | --- | --- | --- | --- | --- |
| 5 | SPVLRSHSF | SPVSRSHSF | 9 | HLA-B*35:01 | 0.831 | 0 | 0.035 | 0.047 | 32.086 | 0.980 | 0.024 |
| 5 | YPVIFKSIM | YPVISKSIM | 9 | HLA-B*35:01 | 1.065 | 1 | 0.083 | 0.304 | 46.265 | 0.883 | 0.024 |
| 5 | QPPALSPSY | QPPAPSPSY | 9 | HLA-B*35:01 | 0.741 | 0 | 0.042 | 0.019 | 32.877 | 0.758 | 0.031 |
| 5 | APSPGQPPAL | APSPGQPPAP | 10 | HLA-B*35:01 | 0.014 | 0 | 0.689 | 0.620 | 88.769 | 0.813 | 0.032 |
| 5 | ALPQSILLF | ALPRSILLF | 9 | HLA-A*23:01 | 0.491 | 0 | 0.146 | 0.299 | 89.793 | 0.707 | 0.036 |
| 5 | NEYAFVTTF | DEYAFVTTF | 9 | HLA-B*41:02 | 0.018 | 0 | 0.089 | 0.185 | 48.378 | 0.981 | 0.053 |
| 5 | QGLPNEYAF | QGLPDEYAF | 9 | HLA-A*23:01 | 0.394 | 0 | 1.436 | 1.022 | 1019.570 | 0.096 | 0.068 |
| 5 | YGIRGFSTI | YGIRGFPTI | 9 | HLA-A*23:01 | 1.513 | 0 | 3.600 | 1.019 | 3697.969 | 0.041 | 0.073 |
| 5 | TALPQSILLF | TALPRSILLF | 10 | HLA-B*35:01 | 0.177 | 0 | 1.341 | 0.605 | 1754.038 | 0.128 | 0.076 |
| 5 | KEYPVIFKSI | KEYPVISKSI | 10 | HLA-B*41:02 | 0.790 | 0 | 0.510 | 0.239 | 55.044 | 0.923 | 0.077 |
| 5 | FSPGAANLF | SSPGAANLF | 9 | HLA-A*23:01 | 0.708 | 0 | 1.141 | 2.536 | 318.789 | 0.275 | 0.084 |
| 5 | LPNEYAFVTT | LPDEYAFVTT | 10 | HLA-B*35:01 | 0.375 | 1 | 4.521 | 0.480 | 2294.769 | 0.232 | 0.085 |
| 5 | REFDKIELA | REFDEIELA | 9 | HLA-B*41:02 | 1.508 | 0 | 0.075 | 0.102 | 39.440 | 0.980 | 0.089 |
| 5 | LPNEYAFVT | LPDEYAFVT | 9 | HLA-B*35:01 | 0.010 | 1 | 0.923 | 0.707 | 221.832 | 0.549 | 0.090 |
| 5 | REFDKIELAY | REFDEIELAY | 10 | HLA-B*41:02 | 0.940 | 1 | 0.527 | 0.339 | 85.430 | 0.936 | 0.139 |
| 5 | DYVSALHPV | DHVSALHPV | 9 | HLA-A*23:01 | 0.032 | 0 | 1.792 | 1.914 | 751.014 | 0.164 | 0.178 |
| 5 | MTITSRGTTV | MTITPRGTTV | 10 | HLA-A*66:01 | 0.123 | 0 | 5.016 | 1.555 | 8128.320 | 0.015 | 0.237 |
| 5 | IFKSIMRQRL | ISKSIMRQRL | 10 | HLA-A*23:01 | 0.277 | 0 | 2.445 | 0.911 | 4404.222 | 0.033 | 0.240 |
| 5 | HTHYDYVSAL | HTHYDHVSAL | 10 | HLA-A*66:01 | 0.003 | 0 | 0.894 | 2.475 | 6586.362 | 0.034 | 0.339 |
| 5 | YDYVSALHPV | YDHVSALHPV | 10 | HLA-B*41:02 | 0.336 | 0 | 5.988 | 23.659 | 5031.145 | 0.019 | 0.649 |
| 6 | DMKARQKAL | DMKARQEAL | 9 | HLA-B*08:01 | 0.526 | 0 | 0.004 | 0.002 | 34.720 | 0.919 | 0.000 |

Supplementary Table 8 Continued

| Patient | Peptide Mut | Peptide Wild | Pep Length | HLA | Agretopicity | Hit | NetMHCpan4.1 | MixMHCpred2.2 | MHCflurry2.0 BA | MHCflurry2.0 PS | NeoMUST |
| --- | --- | --- | --- | --- | --- | --- | --- | --- | --- | --- | --- |
| 6 | WTNCILHEY | WTNRILHEY | 9 | HLA-A*01:03 | 1.349 | 0 | 0.160 | 0.079 | 67.189 | 0.787 | 0.004 |
| 6 | ISDRFIGIY | ISDRLIGIY | 9 | HLA-A*01:03 | 1.743 | 0 | 0.038 | 0.023 | 41.679 | 0.948 | 0.005 |
| 6 | LLDKFVEWY | LLDEFVEWY | 9 | HLA-A*01:03 | 1.375 | 0 | 0.100 | 0.017 | 46.372 | 0.899 | 0.005 |
| 6 | ESIKEITNF | ESIKEITNL | 9 | HLA-A*66:01 | 0.051 | 0 | 0.177 | 0.301 | 204.732 | 0.811 | 0.010 |
| 6 | ESIKEITNFK | ESIKEITNLK | 10 | HLA-A*66:01 | 0.214 | 0 | 0.078 | 0.436 | 66.102 | 0.827 | 0.012 |
| 6 | SIKEITNFK | SIKEITNLK | 9 | HLA-A*66:01 | 0.148 | 0 | 0.110 | 0.872 | 80.869 | 0.831 | 0.012 |
| 6 | VVDFKKNLEY | VVDLKKNLEY | 10 | HLA-A*01:03 | 0.063 | 0 | 0.043 | 0.023 | 48.660 | 0.778 | 0.015 |
| 6 | EISDRFIGIY | EISDRLIGIY | 10 | HLA-A*66:01 | 0.014 | 0 | 0.218 | 10.655 | 221.270 | 0.770 | 0.025 |
| 6 | QTSIQSPSLY | QTSIQSPSSY | 10 | HLA-A*01:03 | 0.519 | 0 | 0.072 | 0.015 | 75.529 | 0.572 | 0.025 |
| 6 | NVELRRNVL | NVELPRNVL | 9 | HLA-B*08:01 | 0.930 | 0 | 0.096 | 0.305 | 62.904 | 0.649 | 0.026 |
| 6 | ESDLNSWPV | ESDLDSWPV | 9 | HLA-A*01:03 | 0.741 | 0 | 0.973 | 0.539 | 3238.226 | 0.030 | 0.033 |
| 6 | WTNCILHEY | WTNRILHEY | 9 | HLA-A*66:01 | 0.741 | 0 | 0.756 | 2.458 | 277.344 | 0.482 | 0.041 |
| 6 | HTLGAASSF | HTLGAASPF | 9 | HLA-A*66:01 | 0.008 | 0 | 0.317 | 0.604 | 170.290 | 0.530 | 0.044 |
| 6 | TSIQSPSLY | TSIQSPSSY | 9 | HLA-A*01:03 | 0.044 | 0 | 0.077 | 0.074 | 91.419 | 0.579 | 0.046 |
| 6 | HLARHRHLM | HPARHRHLM | 9 | HLA-B*08:01 | 0.948 | 0 | 0.159 | 1.106 | 62.448 | 0.588 | 0.051 |
| 6 | DMKARQKALV | DMKARQEALV | 10 | HLA-B*08:01 | 1.687 | 1 | 0.843 | 1.436 | 3833.246 | 0.035 | 0.058 |
| 6 | LPVTRKNMPL | LPVTRKNMPP | 10 | HLA-B*08:01 | 2.224 | 0 | 0.851 | 2.647 | 135.196 | 0.413 | 0.072 |
| 6 | EISDRFIGI | EISDRLIGI | 9 | HLA-A*66:01 | 1.223 | 0 | 0.652 | 1.740 | 436.654 | 0.742 | 0.076 |
| 6 | QTRKNKKLAL | QTRKNKKLAP | 10 | HLA-B*08:01 | 0.918 | 0 | 0.644 | 3.918 | 397.931 | 0.196 | 0.105 |
| 6 | QLFARARPM | RLFARARPM | 9 | HLA-B*08:01 | 0.023 | 0 | 0.343 | 0.981 | 74.061 | 0.553 | 0.111 |
| 6 | HTLGAASSFM | HTLGAASPFM | 10 | HLA-A*66:01 | 0.407 | 0 | 2.321 | 0.844 | 283.623 | 0.252 | 0.120 |

Supplementary Table 8 Continued

| Patient | Peptide Mut | Peptide Wild | Pep Length | HLA | Agretopicity | Hit | NetMHCpan4.1 | MixMHCpred2.2 | MHCflurry2.0 BA | MHCflurry2.0 PS | NeoMUST |
| --- | --- | --- | --- | --- | --- | --- | --- | --- | --- | --- | --- |
| 6 | SPHLARHRHL | SPHPARHRHL | 10 | HLA-B*08:01 | 1.259 | 0 | 0.352 | 9.852 | 616.430 | 0.222 | 0.194 |
| 6 | EMKRVFGFPV | EMERVFGFPV | 10 | HLA-B*08:01 | 0.561 | 0 | 14.333 | 7.741 | 19497.265 | 0.005 | 0.217 |
| 6 | SSQPQVPFY | SSQPQVPFH | 9 | HLA-A*01:03 | 1.020 | 0 | 0.051 | 0.039 | 92.073 | 0.675 | 0.262 |

**Supplementary Table 8. TeSet-4 Data Set with the Prediction Results.** A higher score from the MHCflurry2.0 PS predictor indicates a better chance of successfully presenting neoantigens, while lower scores from other predictors suggest an increased likelihood of successful neoantigen presentation.
