# Supplementary material for: NeoMUST: an accurate and efficient multi-task learning model for neoantigen presentation
Source: Life Sci Alliance. 2024 Jan 30;7(4):e202302255. doi: 10.26508/lsa.202302255 (PMC10828515; doi:10.26508/lsa.202302255)
Supplement: Supplementary file 13 [file LSA-2023-02255_TableS9.docx]

# 8 Supplementary Table 9

| Models/Parameters | Input | Specific Experts | Shared Experts | Experts Hidden | Experts Out | Towers Hidden |
| --- | --- | --- | --- | --- | --- | --- |
| NeoMUST-1 | 256 | 1 | 1 | 128 | 64 | 32 |
| NeoMUST-1-Tiny | 256 | 1 | 1 | 64 | 16 | 4 |
| NeoMUST-2 | 256 | 2 | 2 | 128 | 64 | 32 |
| NeoMUST-2-Tiny | 256 | 2 | 2 | 64 | 16 | 4 |
| NeoMUST-3 | 256 | 3 | 3 | 128 | 64 | 32 |
| NeoMUST-3-Tiny | 256 | 3 | 3 | 64 | 16 | 4 |
| NeoMUST-4 | 256 | 4 | 4 | 128 | 64 | 32 |
| NeoMUST-4-Tiny | 256 | 4 | 4 | 64 | 16 | 4 |
| NeoMUST-5 | 256 | 5 | 5 | 128 | 64 | 32 |
| NeoMUST-5-Tiny | 256 | 5 | 5 | 64 | 16 | 4 |

**Supplementary Table 9. Detailed parameters of NeoMUST ensemble model.**
