# Supplementary material for: NeoMUST: an accurate and efficient multi-task learning model for neoantigen presentation
Source: Life Sci Alliance. 2024 Jan 30;7(4):e202302255. doi: 10.26508/lsa.202302255 (PMC10828515; doi:10.26508/lsa.202302255)
Supplement: Supplementary file 14 [file LSA-2023-02255_TableS10.docx]

# 10 Supplementary Table 10

| **Software and Models / Benchmark DataSet** | **Neoantigen Presentation** | | | | **Binding Affinity** | **Immunogenicity** |
| --- | --- | --- | --- | --- | --- | --- |
|  | TeSet-1 | TeSet-1-Filtered | TeSet-2 | TeSet-2-Filtered | TeSet-3 | TeSet-4 |
| NetMHCpan4.0 EL | √ |  | √ |  |  |  |
| NetMHCpan4.1 EL |  | √ |  | √ |  | √ |
| NetMHCpan4.1 BA |  |  |  |  | √ |  |
| MixMHCpred2.2 |  | √ |  | √ |  | √ |
| MHCflurry2.0 BA | √ |  | √ |  | √ | √ |
| MHCflurry2.0 PS | √ |  |  |  |  | √ |
| NeoMUST NP | **√** | **√** | **√** | **√** |  | **√** |
| NeoMUST BA |  |  |  |  | **√** |  |
| NeoMUST_ensemble NP | **√** |  | **√** |  |  |  |
| NeoMUST_drop_BA NP | **√** |  |  |  |  |  |

**Supplementary Table 10. Summary of the utilization of benchmark models and datasets.**
